# ALTERNATIVE SPLICING DYNAMICS IN MYOGENESIS: A TRANSCRIPTOMIC ATLAS FOR HUMAN SKELETAL MUSCLE CELLS

**DOI:** 10.1093/geroni/igae098.2817

**Published:** 2024-12-31

**Authors:** Stefano Donega, Nirad Banskota, Jen-Hao Yang, Martina Rossi, Dimitrios Tsitsipatis, Supriyo De, Myriam Gorospe, Luigi Ferrucci

**Affiliations:** National Institute on Aging, Baltimore, Maryland, United States; National Institutes of Health, Bethesda, Maryland, United States; National Institutes of Health, Bethesda, Maryland, United States; National Institutes of Health, Bethesda, Maryland, United States; National Institute on Aging, Baltimore, Maryland, United States; National Institute on Aging, Baltimore, Maryland, United States; National Institutes of Health, Bethesda, Maryland, United States; National Institute on Aging, Baltimore, Maryland, United States

## Abstract

Myogenesis entails the fusion of myoblasts to form multinucleated myotubes. Although alternative splicing (AS) in myogenesis has been explored in non-human species, its characterization in humans remains incomplete, both in vitro and in vivo. We studied two human skeletal muscle cell lines (AB1167 and AB678) over a 5-day differentiation period, with RNA samples collected every 24 hours. Utilizing a Hybrid RNA-sequencing approach (combining short and long-read sequencing), we applied linear regression in Differential Gene Expression (DGE) and Likelihood Ratio Test – Hierarchical Clustering models (LRT-HCl), and discovered 3,079 upregulated genes and 2,357 downregulated genes in the overlapping models. Gene Set Enrichment Analysis (GSEA) highlighted pathways related to muscle formation and contraction showing upregulation, while those associated with nuclear separation and DNA synthesis were downregulated until day 3, followed by an increase in inflammation and interferon-like pathways by day 5. Differential Transcript Usage analysis (DTU) identified 2,584 myogenesis-dependent isoform-switches, which were primarily linked to actin-filament processes and muscle regulation. Validation of the models was conducted for the top 5 genes using qPCR. Additionally, three novel protein-coding genes associated with muscle differentiation were identified through an Alphafold-based computational pipeline and validated via qPCR. Comparison of clusters within a 0–3-day model with a Peripheral Artery Disease (PAD) dataset confirmed significant enrichment of up-regulated clusters observed in the myogenesis model. This project aims to establish a comprehensive transcriptomic atlas for myogenesis models and set a benchmark for future research in the multi-omic fields of muscle biology.

